# *In situ* formation of surface-functionalized ionic calcium carbonate nanoparticles with liquid-like behaviours and their electrical properties

**DOI:** 10.1098/rsos.170732

**Published:** 2018-01-17

**Authors:** Xiangshuo Wang, Ling Shi, Junying Zhang, Jue Cheng, Xiaodong Wang

**Affiliations:** 1 Key Laboratory of Beijing City on Preparation and Processing of Novel Polymer Materials, Beijing University of Chemical Technology, Beijing 100029, People's Republic of China; 2 State Key Laboratory of Organic–Inorganic Composite Materials, Beijing University of Chemical Technology, Beijing 100029, People's Republic of China

**Keywords:** nanoscale ionic materials, CaCo_3_, *in situ* formation, liquid-like behaviours, electrical properties

## Abstract

This paper reports a new route to synthesize calcium carbonate (CaCO_3_)-based nanoscale ionic materials (NIMs) via an *in situ* formation method to form the CaCO_3_ nanoparticles with a polysiloxane quaternary ammonium salt (PQAC) corona (PQAC-CaCO_3_ nanoparticles), followed by an ionic exchange reaction to fabricate a poly(ethylene glycol)-tailed sulfonate anion (NPEP) canopy. The chemical compositions and structures of the CaCO_3_-based NIMs synthesized in this work were confirmed by Fourier-transform infrared spectroscopy and solid-state ^13^C NMR spectroscopy. Transmission electron microscopic observation indicated that the CaCO_3_-based NIMs presented a rhombohedral shape with a well-defined core-shell structure, and they also obtained an NPEP canopy with a thickness of 4–6 nm. X-ray powder diffraction investigation confirmed that the CaCO_3_ inner core had a calcite crystalline structure, whereas the NPEP canopy was amorphous. The NPEP canopy was found to show a characteristic crystallization–melting behaviour in the presence of the ion bonding with PQAC-CaCO_3_ nanoparticles according to the characterization of differential scanning calorimetry. Thermogravimetric analysis indicated that the CaCO_3_-based NIMs achieved a high content of NPEP canopy as well as an improvement in thermal stability owing to the ion-bonding effect. Most of all, the CaCO_3_-based NIMs demonstrated a liquid-like behaviour above the critical temperature in the absence of solvent. Moreover, the CaCO_3_-based NIMs also showed a relatively high electrical conductivity with a temperature dependency due to the ionic conductive effect. This work will provide a more feasible and energy-saving methodology for the preparation of CaCO_3_-based NIMs to promote their industrialization and extensive applications.

## 1. Introduction

Nanoscale ionic materials (NIMs) are a type of nanoparticles with liquid-like behaviour in the absence of solvent, which were pioneered by Giannelis *et al.* [1]. With unique rheological properties and other special functions attributed to distinct properties of different nanoparticles, NIMs have attracted extensive attention from scientific societies to industrial communities in recent years [2]. NIMs are typically composed of an inorganic nanosized core, a covalently tethered ionic corona and an ionically tethered oligomeric canopy, and therefore they have a core–corona–canopy structure [3]. Such a structural feature leads to homogeneous fluids rather than a dual-phase dispersion of nanoparticles suspended in organic liquids and also makes NIMs achieve flow properties that span the range from glassy solids to free-flowing liquids [4], and hence, their solvent-free fluidity with zero vapour pressure offers a new prospect in scientific and technological applications. In general, NIMs have a number of fantastic characteristics such as a wide liquid temperature scope up to 250°C, a wide range of dissolution and dispersion with various organic, inorganic, organometallic compounds and polymers, a wide electrochemical window, zero vapour pressure, good ionic electrical conduction, non-volatility, non-polluting and eco-friendly [5]. Therefore, the formation of NIMs not only can overcome some of the challenges facing nanocomposite materials such as the aggregation and agglomeration of nanoparticles owing to their stable monodispersity and very low viscosity at room temperature, but also can endow them with various new properties derived from their diverse inorganic cores [6–10]. Nowadays, NIMs have become a centrepiece for the design platform, which endows the nanomaterials with versatility and a unique set of properties and opens up a wide range of applications. NIMs have been broadly applied as functional ionic liquids [11], liquid CO_2_ absorbents [12], highly lubricative dispersions [13] and functional coatings with controlled surface properties [14], and they can also be used as electrolytes for lithium-ion batteries [15], precursor materials for highly porous separation films [16] and good candidate materials for various sensors [17].

It is widely recognized that the flowing capability or so-called ‘liquid-like behaviour' of NIMs is derived from the effective surface functionalization of the targeted core nanostructure with a soft shell through covalent or/and ion-exchange grafting methods, thus providing enough ‘solvent' for the nanoparticles after melting [18]. These solvent-free surface-functionalized nanoparticles are evidently different from those in a polymer matrix, because the surrounding environment consists of other surface-functionalized nanoparticles in solvent-free conditions. With a sufficiently high surface-grafting density, the macromolecular chains of the canopy keep the nanoparticles well dispersed, and hence the nanoparticles can only interact with the neighbouring ones via their overlapping canopy [6]. Therefore, to achieve a liquid-like feature, a high organic content, good plasticizing effect by the organic modifier, weak electrostatic interactions, small size, low density and surface properties of inorganic cores all play key roles in isolating a modified nanostructure in liquid form [19–21]. The synthesis of NIMs was reported to include three steps, i.e. the synthesis of nanoparticles, the surface grafting of charged groups to the nanoparticles and the ionic coupling of a flexible organic canopy to the surface-grafted nanoparticles [3]. By now, there have been a number of successful cases for the preparation of NIMs with typical liquid-like behaviours. Giannelis *et al*. [1] first reported the liquid-like properties of the SiO_2_ nanoparticles surface-grafted with silicon alkoxide containing quaternized ammonium groups as a corona and sulfonate anion as a canopy. Warren *et al*. [22] developed a generalized route to prepare a type of platinum-based NIMs by surface-grafting organic bromide as a corona and sulfonate anion as a canopy and found that this type of metallic NIMs exhibited a good fluidity at room temperature, whereas pure platinum could flow only above the melting point of 1000°C. Moganty *et al*. [11] reported a new class of solvent-free electrolytes with liquid-like behaviours, and these electrolytes could be fabricated by tethering ionic liquids to hard inorganic ZrO_2_ nanostructures. Sun *et al*. [23] reported the synthesis and liquid-like behaviours of colloidal PbS quantum dots by protonating sodium mercaptopropanesulfonate as a corona, followed by neutralizing with polyetheramine as a canopy. Bourlinos *et al*. [24] reported a type of one-dimensional NIMs consisting of carboxylic carbon nanotubes surface-grafted with poly(ethylene glycol)-substituted tertiary amine, and this type of NIMs demonstrated a reversible solid–liquid transition at 35°C. Li *et al*. [25] reported a novel type of NIMs based on graphene sheets surfaced-grafted with aryl diazonium salt and oligomeric quaternary ammonium salt as a corona and canopy, respectively, and found that these two-dimensional NIMs behaved like a molten salt at room temperature.

Calcium carbonate (CaCO_3_) is one of the most abundant inorganic materials in the earth and has achieved broad applications as a type of inorganic filler in various industrial fields [26,27]. As a type of effective functional additive or filler for high-performance organic–inorganic nanocomposites, CaCO_3_ nanoparticles have attracted great attention from academic communities and industrial societies owing to their high surface activity, good reinforcing and whitening effects, low cost and easy availability [28]. However, CaCO_3_ nanoparticles are easy to agglomerate because of their high surface energy, which makes them difficult to be dispersed in the polymeric matrix when the composites were processed [29]. In this case, the development of CaCO_3_-based NIMs with liquid-like behaviours is of great significance for the extension of their application areas. Until now, liquid-like NIMs composed of CaCO_3_ nanoparticles have only been reported by Li *et al*. [30]. They synthesized a solvent-free nanofluid based on the rhombohedral CaCO_3_ nanoparticles through surface-grafting with a charged polysiloxane quaternary ammonium salt (PQAC), followed by a reaction with sulfonate salts, and found that such a surface functionalization methodology endowed CaCO_3_ nanoparticles with a number of advantageous characteristics such as excellent dispersibility, high fluidity, enhanced processability and electrical conduction. However, as described in their work, the surface functionalization of CaCO_3_ nanoparticles could only be successfully implemented under a strictly controlled acidity of pH 10.5, which has posed a great difficulty for the industrial production of CaCO_3_-based NIMs.

In the current work, we reported a facile synthetic methodology for CaCO_3_-based NIMs through *in situ* formation of liquid-like CaCO_3_ nanoparticles. In this methodology, the CaCO_3_ nanoparticles with a PQAC corona were directly synthesized at first via a carbonation pathway in lime solutions, and then the resultant surface-functionalized CaCO_3_ nanoparticles were further fabricated with a sulfonate salt canopy through an ionic exchange method to achieve the final CaCO_3_-based NIMs. This synthetic route is more succinct and more energy-saving compared to the traditional surface modification of CaCO_3_ nanoparticles. The CaCO_3_-based NIMs developed by this work are expected to demonstrate a solvent-free fluid feature owing to the formation of ionic coupling organic dual-layered CaCO_3_ nanoparticles. The microstructure and morphology of CaCO_3_-based NIMs were characterized in detail, and their liquid-like behaviours and electrical properties were intensively investigated. The aim of this work is to develop a more feasible method for the preparation of CaCO_3_-based NIMs so as to promote their industrialization and extensive applications.

## 2. Experimental section

### 2.1. Material and methods

Carbon dioxide was purchased from Beijing source gas Co. Ltd., China. CaO was purchased from Sinopharm Chemical Reagent Co. Ltd., China. Poly(ethylene glycol) 4-nonylphenyl 3-sulfopropyl ether potassium salt (NPEP, C_9_H_19_C_6_H_4_O(CH_2_CH_2_O)_10_SO_3_^−^K^+^) was commercially obtained from Sigma-Aldrich, USA. A solution of dimethyloctadecyl [3-(trimethoxysilyl) propyl] ammonium chloride [PQAC, (CH_3_O)_3_Si(CH_3_)_3_N^+^(CH_3_)_2_(C_10_H_21_)] in methanol with a volume fraction of 60 vol% was commercially supplied by Acros Organics, Belgium. The other chemicals were purchased from Beijing Chemical Reagent Co. Ltd., China and used as received without additional purification.

### 2.2. Synthesis

The CaCO_3_ nanoparticles with a PQAC corona (PQAC-CaCO_3_ nanoparticles) were first prepared through an *in situ* formation method, and the carbonation experiments were carried out in a bubble column at 13–15°C. A CO_2_/air mixed gas (vol/vol = 1/3) was bubbled into the Ca(OH)_2_ slurry (30–35 wt%) with a flowing rate of 6–7 ml s^−1^ to produce CaCO_3_ nanoparticles. The carbonation reaction is stopped as soon as the acidity declined up to pH 7.0, and then PQAC was added into the resultant suspension to conduct a surface-grafting reaction among the hydrogen groups on CaCO_3_ nanoparticles and PQAC at room temperature. The PQAC-CaCO_3_ nanoparticles were obtained after ageing for 20 h. The suspension containing PQAC-CaCO_3_ nanoparticles was filtered, washed and then dried at 110°C for 24 h.

The PQAC-CaCO_3_ nanoparticles were further chemically grafted with an NPEP canopy through an ionic exchange between the chloride anions on PQAC and the sulfonate anions on NPEP. In a typical process, an amount of 10.0 g of PQAC-CaCO_3_ nanoparticles was added into 150 ml of NPEP aqueous solution (10.5 wt%), and the ionic exchange reaction was performed at 70°C for 24 h. The CaCO_3_-based NIMs were obtained by removal of the solvent and washing with water [1].

### 2.3. Characterization

The microstructure of CaCO_3_-based NIMs was identified by transmission electron microscopy (TEM) using a Tecnai G2 20 transmission electron microscope. The bright field TEM images were obtained at 200 kV, and the selected area electron diffraction (SAED) pattern was achieved along with the TEM observation of CaCO_3_-based NIMs. X-ray powder diffraction (XRD) was conducted on a Rigaku Ultima IV X-ray diffractometer with a Cu–K*α* radiation (*λ *= 1.5405 Å). Fourier-transform infrared (FTIR) spectroscopy was performed on a Bruker Alpha-T FTIR spectrometer at a scanning number of 32. The solid-state ^13^C NMR spectrum of CaCO_3_-based NIMs was obtained on a Bruker AV-300 300 MHz spectrometer. Differential scanning calorimetry (DSC) was performed by using a TA Instruments Q20 differential scanning calorimeter in nitrogen at a scanning rate of 10°C min^−1^. Thermogravimetric analysis (TGA) was performed on a TA Instruments Q50 thermogravimetric analyser at a heating rate of 10°C min^−1^ under a nitrogen atmosphere. TG–FTIR spectroscopy coupled analysis was conducted on a Mettler Toledo TGA/DSC 1 STARe System coupled with a Nicolet 6700 FTIR spectrometer equipped with an infrared gas cell. The rheological properties of CaCO_3_-based NIMs were investigated by using a TA DHR-1 rheometer. The modulus G′ and G′ were measured at an angular frequency of 1 HZ with a strain amplitude of 50% in the temperature range from 0 to 70°C. The ionic conductivity of CaCO_3_-based NIMs was measured on a broad-band dielectric spectrometer (Novocontrol Concept 50, Germany).

## 3. Results and discussion

**Figure 1.**
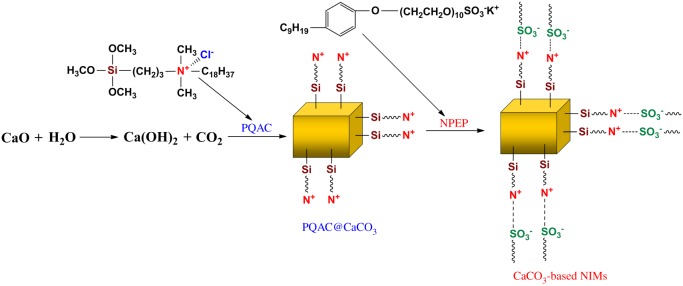
Schematic route for the synthesis of CaCO_3_-based NIMs.

**Figure 2.**
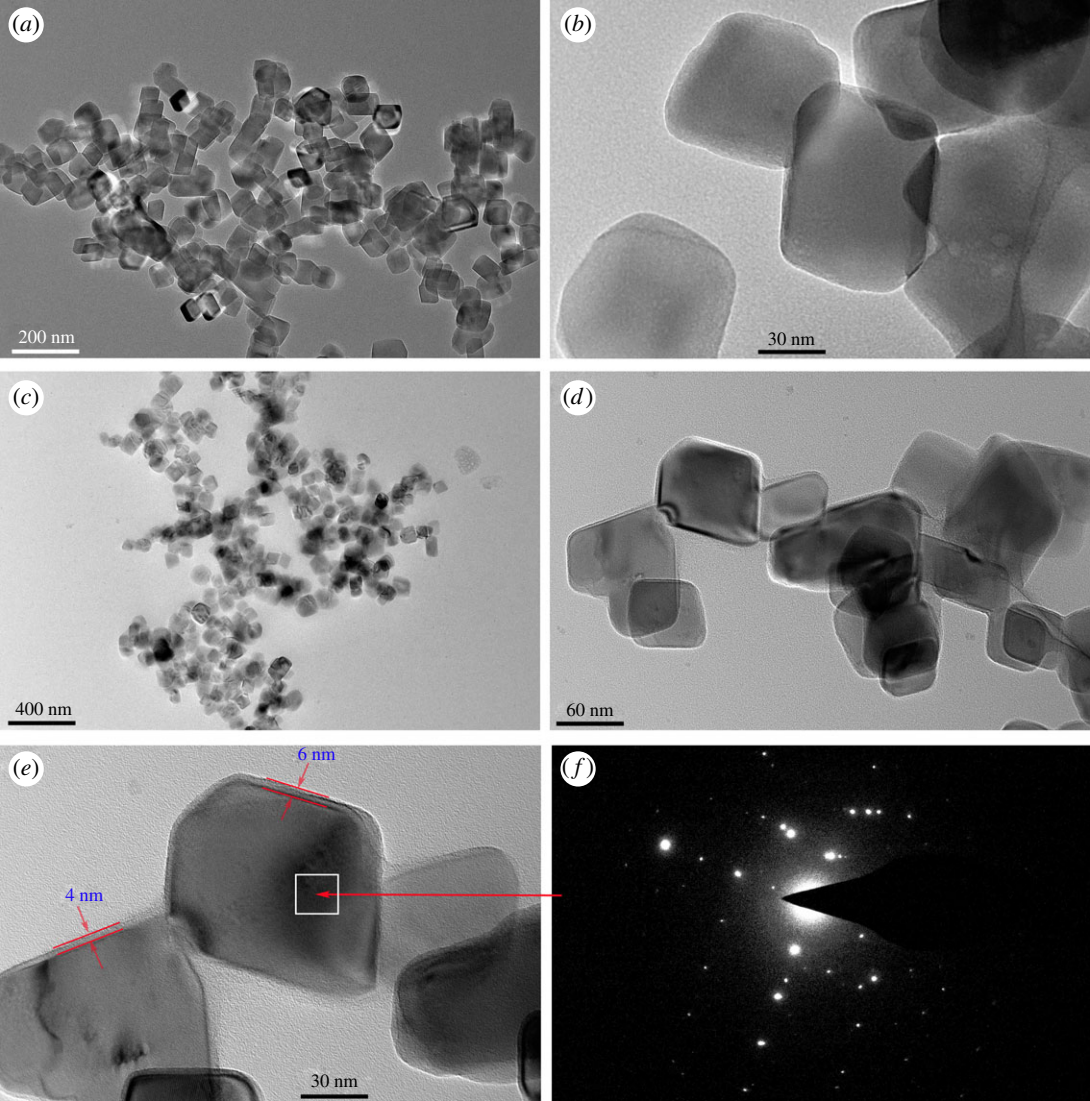
TEM micrographs of (*a*) PQAC-CaCO_3_ nanoparticles, (*b*) zoomed-in structure of PQAC-CaCO_3_ nanoparticles, (*c*) CaCO_3_-based NIMs, (*d*,*e*) zoomed-in structure of CaCO_3_-based NIMs and (*f*) SAED pattern of CaCO_3_-based NIMs.

The preparation of CaCO_3_-based NIMs was carried out via an *in situ* formation method, and its synthetic route was schematically demonstrated in figure 1. This synthesis looks straightforward and just requires the *in situ* formation of PQAC-CaCO_3_ nanoparticles followed by an ion exchange reaction of the PQAC corona with NPEP to form a macromolecular canopy. In a typical formation procedure as shown in figure 1, CaCO_3_ nanoparticles were first prepared by a reaction of Ca(OH)_2_ with CO_2_ in a bubble column, and the synchronous surface functionalization was performed through a condensation reaction between the hydroxyl groups on the surface of CaCO_3_ nanoparticles and the silanol groups of PQAC. With the formation of a PQAC corona, the chloride counterions are presented on the surface of CaCO_3_ nanoparticles to be readily exchanged with other anions. In this case, a macromolecular canopy could be facilely fabricated onto the PQAC-CaCO_3_ nanoparticles through an ionic exchange reaction between the chloride anions on the PQAC-CaCO_3_ nanoparticles and the sulfonate anions of NPEP to obtain the CaCO_3_-based NIMs. Figure 2 shows the TEM micrographs of PQAC-CaCO_3_ nanoparticles and CaCO_3_-based NIMs. The PQAC-CaCO_3_ nanoparticles are observed to present a regular rhombohedral shape with a homogeneous size of 60–70 nm. It is also notable that CaCO_3_ nanoparticles have been well isolated by the PQAC corona in a monodispersed form. It is interestingly observed from figure 2*c,e* that, after capping the PQAC-CaCO_3_ nanoparticles with an NPEP canopy, the resulting CaCO_3_-based NIMs exhibit a well-defined core-shell nanostructure, in which a thin outer layer with comparably low contrast can be distinguished to sheath a denser inner core [31]. Such an outer layer is found to have a thickness of 4–6 nm and is considered as the NPEP canopy. Moreover, The CaCO_3_-based NIMs are also observed still keeping intact after the ion exchange reaction of PQAC-CaCO_3_ nanoparticles with NPEP. In addition, the SAED pattern of the CaCO_3_ inner core in figure 2*f* displays very sharp diffraction spots, indicating a single-crystalline nature with a high crystallographic orientation [32].

**Figure 3.**
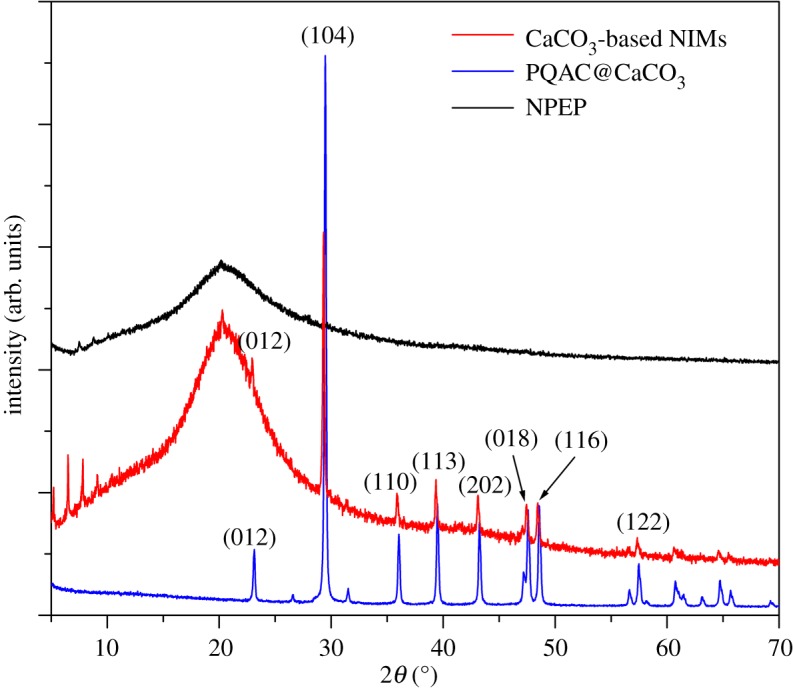
XRD patterns of CaCO_3_-based NIMs, PQAC-CaCO_3_ nanoparticles and pristine NPEP.

Figure 3 shows the XRD patterns of PQAC-CaCO_3_ nanoparticles and CaCO_3_-based NIMs as well as pristine NPEP as a reference. In the XRD pattern of PQAC-CaCO_3_ nanoparticles, a series of intensive diffraction peaks can be observed at 2*θ* = 23.0°, 29.4°, 36.0°, 39.4°, 43.1°, 47.5°, 48.5° and 57.4°, which are well assigned to the (012), (104), (110), (113), (202), (018), (116) and (122) planes of CaCO_3_, respectively [33]. These diffraction data clearly indicate that the CaCO_3_ nanoparticles are completely formed by rhombohedral crystals and also confirm a calcite structure according to the JCPDS card no. 05-0586. On the other hand, pristine NPEP only shows a broad peak centred at 20° in its XRD pattern, suggesting the amorphous nature of NPEP, probably with the presence of some liquid-crystalline domains [18]. It is noteworthy in figure 3 that all of the diffraction peaks from PQAC-CaCO_3_ nanoparticles can be found in the XRD pattern of CaCO_3_-based NIMs, indicating that the intrinsic crystalline structure of the CaCO_3_ inner core is not influenced by the fabrication of the NPEP canopy through an ionic exchange reaction. However, the crystallinity of the NPEP canopy seems to be enhanced owing to the presence of some small diffraction peaks in the XRD pattern of CaCO_3_-based NIMs.

**Figure 4.**
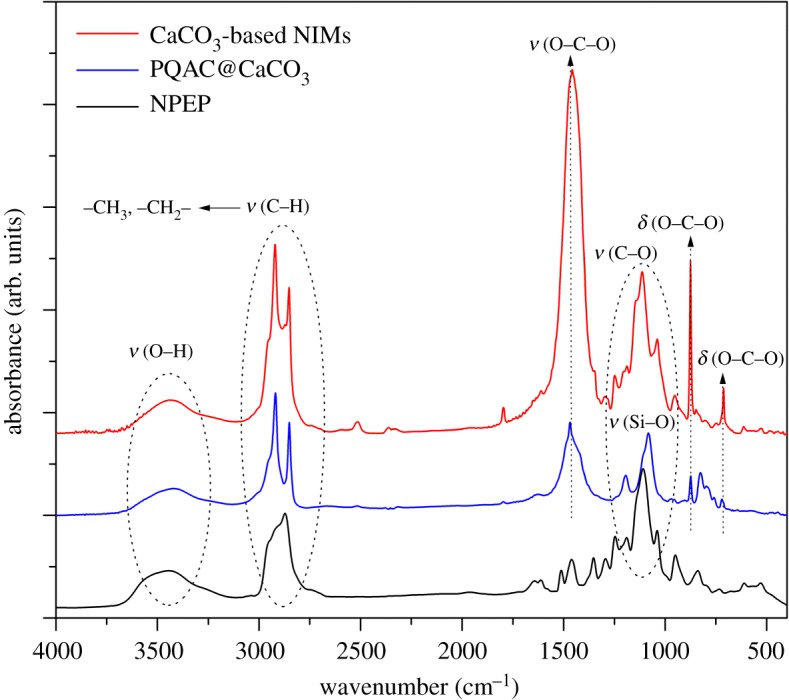
FTIR spectra of CaCO_3_-based NIMs, PQAC-CaCO_3_ nanoparticles and pristine NPEP.

**Figure 5.**
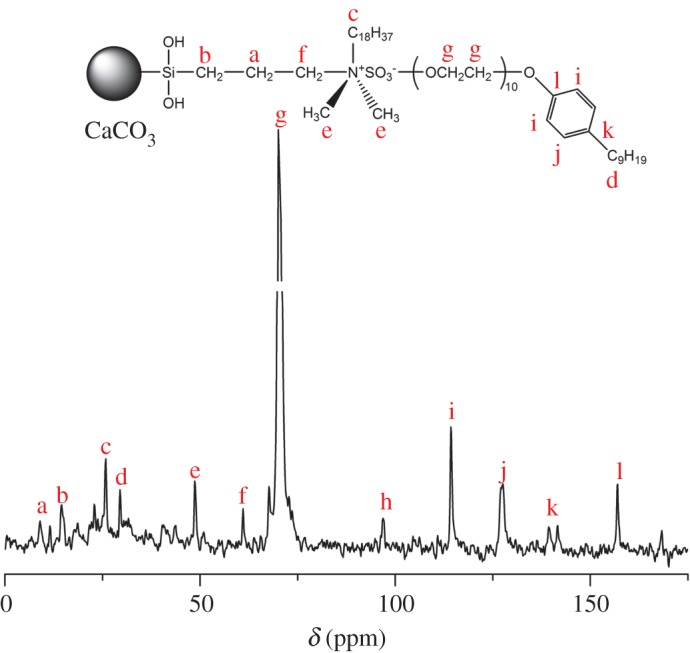
Solid-state ^13^C NMR spectrum of CaCO_3_-based NIMs.

The chemical structures and compositions of PQAC-CaCO_3_ nanoparticles and CaCO_3_-based NIMs were investigated by FTIR spectroscopy, and the obtained infrared spectra were presented in figure 4. As observed from the infrared spectrum of PQAC-CaCO_3_ nanoparticles, two absorption bands are observed at 709 and 874 cm^−1^ which are attributed to the in-plane and out-of-plane bending vibrations of the C–O bond of the carbonate moiety, respectively [34]. This confirms the presence of calcite CaCO_3_. On the other hand, two intensive absorption peaks that appear at 2980 and 2820 cm^−1^ are assigned to the alkyl C–H stretching vibrations of methyl and methylene groups in the PQAC corona, respectively. Moreover, an intensive absorption band can be found at 1079 cm^−1^ owing to the Si–O stretching vibration, which implicates that the hydrolysed PQAC has condensed with the hydroxyl groups on the surfaces of CaCO_3_ nanoparticles. It is noteworthy in figure 4 that the characteristic absorption bands corresponding to CaCO_3_ nanoparticles are all found in the infrared spectrum of CaCO_3_-based NIMs. Furthermore, a series of absorption peaks observed from 1035 to 1248 cm^−1^ are attributed to the asymmetric stretching vibration of the C–O–C band in the glycoside moiety of NPEP as well as the coupled stretching vibrations of C–C and C–O bands in NPEP [35]. Meanwhile, a set of absorption bands corresponding to the skeletal vibrations of the aromatic ring are observed from 1500 to 1600 cm^−1^. These characteristic absorption bands are in good agreement with those appearing in the infrared spectrum of pristine NPEP, thus confirming the successful fabrication of the NPEP canopy. The chemical structures of organic PQAC corona and the NPEP canopy were also characterized by solid-state ^13^C NMR spectroscopy and the obtained spectrum is illustrated in figure 5. It is clearly observed that the solid-state ^13^C NMR spectrum of CaCO_3_-based NIMs shows an intensive resonance at *δ* = 70.06 ppm corresponding to the carbon atoms of the methylene group in the NPEP canopy. The other resonance signals are also well assigned to the carbon atoms in the PQAC corona and the NPEP canopy as marked in figure 5. These results further confirmed the successful fabrication of CaCO_3_-based NIMs.

**Figure 6.**
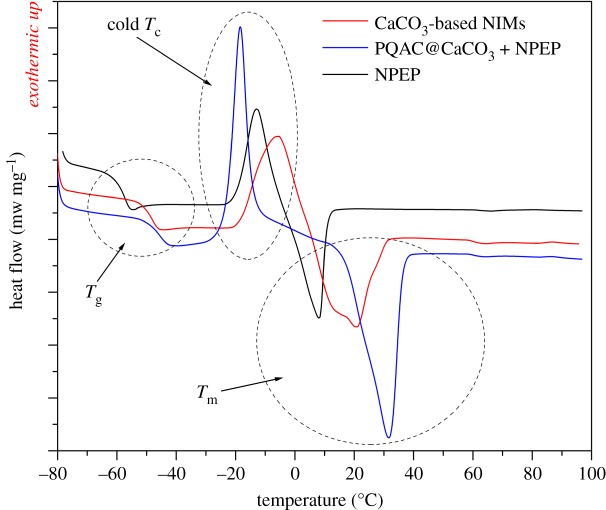
DSC thermograms of CaCO_3_-based NIMs, pristine NPEP and a mixture of PQAC-CaCO_3_ nanoparticles and pristine NPEP.

It was widely reported that pristine NPEP exhibited an interesting crystallization–melting behaviour [1,17,18], although it was usually in the amorphous form as characterized by XRD (figure 3), which might be associated with the liquid-like behaviour. Figure 6 shows the DSC thermograms of CaCO_3_-based NIMs and pristine NPEP, and the thermogram of the mixture of PQAC-CaCO_3_ nanoparticles and pristine NPEP is also presented as a reference. It is observed that the DSC thermogram of pristine NPEP exhibits a glass transition at −58°C followed by a cold crystallization peak at −13°C and then a broad melting peak at 8°C. The CaCO_3_-based NIMs reveal a similar DSC profile with pristine NPEP; however, their glass transition temperatures (*T*_g_) are found to increase to −48°C. This may be owing to the presence of PQAC-CaCO_3_ nanoparticles, which hinders the motivation of NPEP chains. It is interestingly noted from the DSC thermogram of CaCO_3_-based NIMs that both the cold crystallization peak and melting peak shift to higher temperatures. It is understandable that the surface chemical linkage of PQAC-CaCO_3_ nanoparticles with NPEP perturbs the crystallization behaviour of the NPEP canopy significantly, thus resulting in the increase in the cold crystallization temperature. On the other hand, the introduction of CaCO_3_ nanoparticles may generate a heterogeneous nucleation effect for the NPEP canopy and therefore enhances the crystallinity of the NPEP canopy. As a result, the melting temperature of the NPEP canopy is improved. To make a comparative investigation, the thermal behaviour of the mixture of PQAC-CaCO_3_ nanoparticles and pristine NPEP was also analysed by DSC. It is found in figure 6 that the simple mixing of these two matters leads to a decrease in the cold crystallization temperature for pristine NPEP, indicating a significant heterogeneous nucleation effect without the presence of ionic bonding between PQAC-CaCO_3_ nanoparticles and NPEP [7]. Furthermore, such a heterogeneous nucleation effect can promote the perfect crystallization of pristine NPEP and thus leads to a further increase in melting temperature in comparison with the CaCO_3_-based NIMs. These results from DSC analysis clearly demonstrated the characteristic thermal behaviours of NIMs different from those of simple mixtures.

**Figure 7.**
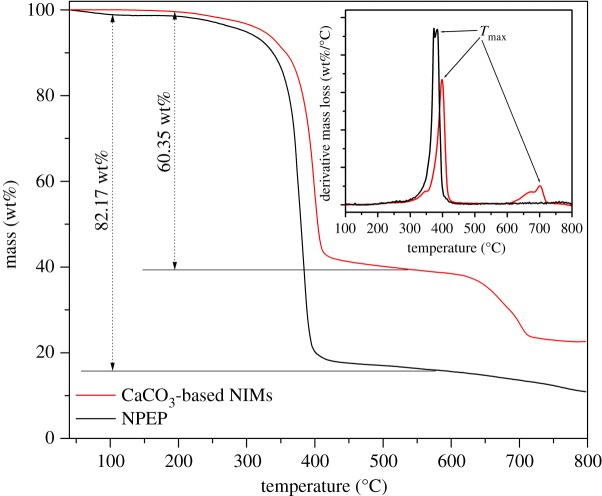
TGA and DTG thermograms of CaCO_3_-based NIMs and pristine NPEP.

**Figure 8.**
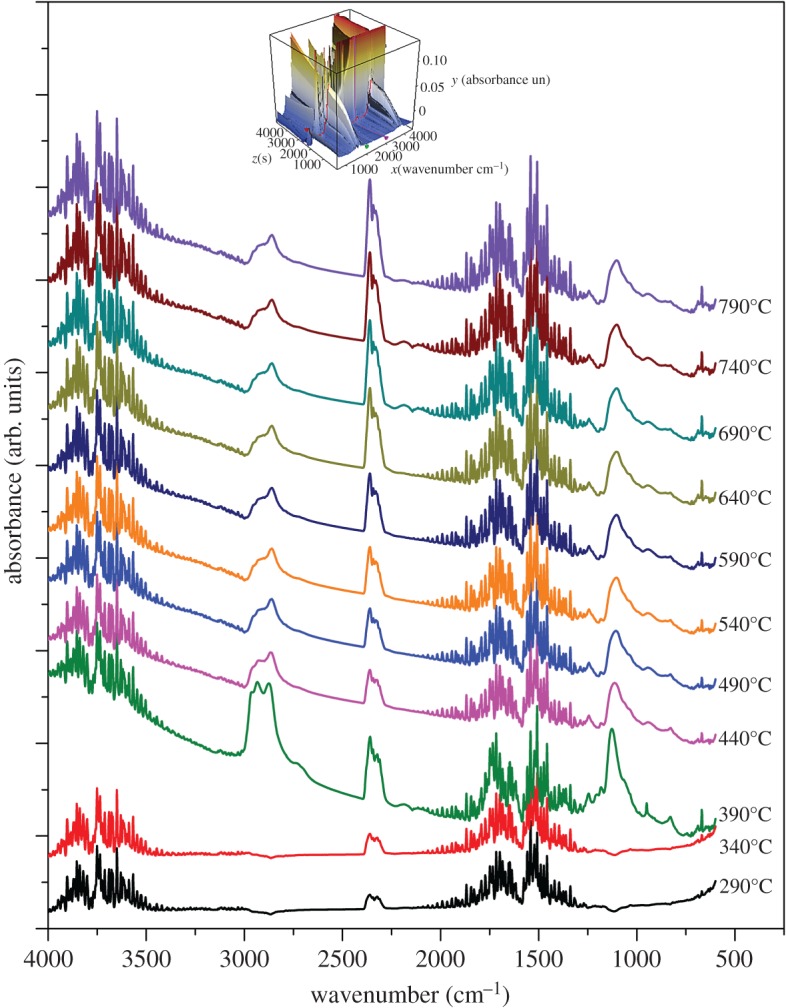
TG-FTIR spectra of CaCO_3_-based NIMs recorded at different temperatures.

The thermal degradation behaviours and thermal stabilities of PQAC-CaCO_3_ nanoparticles and CaCO_3_-based NIMs were evaluated by TGA, and the resulting TGA and derivate TG (DTG) thermograms are presented in figure 7. According to the TGA analysis in figure 7, CaCO_3_-based NIMs exhibit a typical two-step degradation behaviour [1,18], in which the first weight loss occurred at 300–420°C owing to the thermal decomposition of the NPEP canopy and the second one took place at 610–720°C as a result of pyrolysis of the CaCO_3_ inner core. The TGA thermogram also confirms that the CaCO_3_-based NIMs developed by this work are real solvent-free ones, because there is almost no weight loss observed at a temperature below 200°C. On the other hand, pristine NPEP presents a typical one-step thermal decomposition behaviour with a maximum-rate degradation temperature (*T*_max_) of 375°C owing to the pyrolysis of the main chains of NPEP as observed in the inserted DTG thermogram of figure 7. In this case, the content of the NPEP canopy could be determined as 60.35 wt% from the TGA thermogram of CaCO_3_-based NIMs. Although the TEM images in figure 2 only showed a thickness of 4–6 nm for the NPEP canopy, the weight per cent determined by the weight loss in TGA analysis was realistic for the NPEP canopy. The TEM images just presented the dispersed NIM particles, in which the thickness of the macromolecular canopy can be distinguished. However, there are much more aggregated NIM particles which have a thicker canopy layer. Moreover, the macromolecular canopy has much higher density than the CaCO_3_ core, so the weight per cent of 60.35 wt% for the NPEP canopy is believable. Such a high concentration of the macromolecular canopy can effectively provide a solvent-free fluidity for CaCO_3_-based NIMs [20,36]. It is noteworthy that the *T*_max_ of CaCO_3_-based NIMs at the first stage is slightly higher than that of pristine NPEP, suggesting that the fabrication of the NPEP canopy can improve the thermal stability of CaCO_3_-based NIMs owing to the formation of ion-bonding between PQAC-CaCO_3_ nanoparticles and NPEP. This result is in good agreement with the DSC analysis mentioned above. The thermal degradation behaviour of CaCO_3_-based NIMs was further identified by TG-FTIR spectroscopy coupled analysis, and figure 8 shows the resulting infrared spectra of the evolved gases recorded at different temperatures during the TGA measurement in nitrogen. All of the infrared spectra are found to exhibit an absorption peak at 2363 cm^−1^ for CO_2_, a broad peak centred at 3750 cm^−1^ for H_2_O and a series of characteristic bands at 2010–1300 cm^−1^ for benzene rings, indicating that the production of CO_2_, H_2_O and aromatic compounds occurs in the whole pyrolysis process of CaCO_3_-based NIMs. According to these infrared spectra, only CO_2_, H_2_O and the gases of aromatic compounds were evolved at thermal decomposition temperatures lower than 390°C. However, a set of characteristic bands at 3000–2780 cm^−1^ and a single absorption peak at 1098 cm^−1^ are observed with a further elevation in temperature, which are attributed to the C–H stretching vibration of methyl and methylene groups and the stretching vibration of the C–O band, respectively. This is ascribed to the scission of the main chains in the NPEP canopy at higher decomposition temperatures. It is noteworthy in figure 8 that the intensity of absorption peaks corresponding to CO_2_ gas seems to increase considerably at a temperature higher than 590°C owing to the decomposition of the CaCO_3_ core. Moreover, the characteristic bands corresponding to the Cl–H stretching vibration are not found in the infrared spectra, suggesting that all of the chloride ions have been exchanged with sulfonate ions.

**Figure 9.**
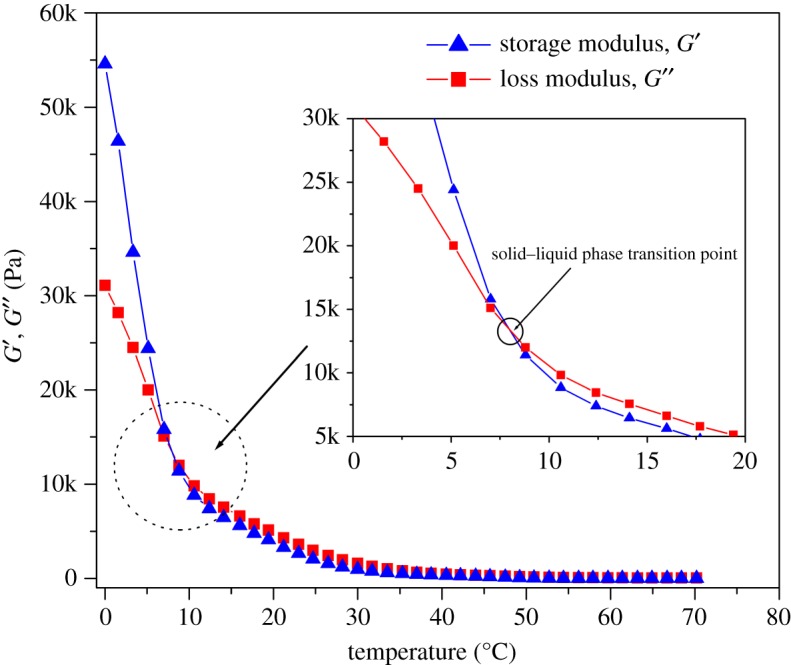
Plots of dynamical storage modulus and loss modulus as a function of temperature for CaCO_3_-based NIMs.

**Figure 10.**
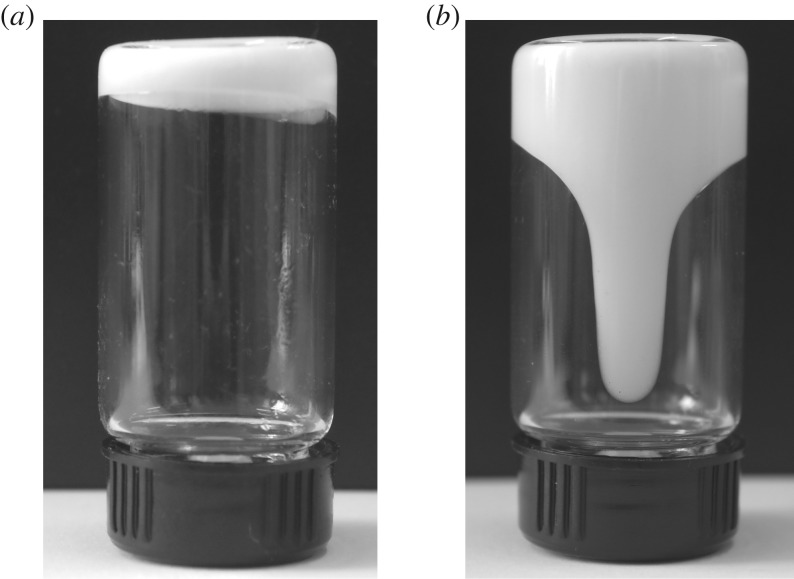
Digital images of a bottom-up bottle CaCO_3_-based NIMs when holding (*a*) at 0°C for 5 min and (*b*) at 23°C for 10 s.

The liquid-like properties of CaCO_3_-based NIMs were investigated by rheological measurements, and the resulting plots of dynamical storage modulus (*G*′) and loss modulus (*G*^″^) as a function of temperature are presented in figure 9. It is well known that both *G*′ and *G*^″^ reveal the relationship between the molecular motion and rheological behaviour of a substance, in which *G*′ denotes the elastic behaviour derived from the driving force for molecule deformation and *G*^″^ represents the consumption energy of viscous deformation for the substance [37]. Therefore, the relationship between the liquid-like behaviours and microstructures of CaCO_3_-based NIMs can be understood from the dynamical rheological spectra as shown in figure 9. CaCO_3_-based NIMs are observed to show a critical temperature at 7.8°C representing a solid–liquid transition in their dynamical rheological spectra. CaCO_3_-based NIMs exhibit a solid behaviour because of *G*′ > *G*^″^ when the temperature is lower than 7.8°C. However, the CaCO_3_-based NIMs demonstrate a liquid-like behaviour as long as the temperature is higher than 7.8°C owing to G′ < G^″^. This is because the liquid substances have permanent deformation and thus exhibits a viscous behaviour. The liquid-like behaviours can also be observed visibly from the digital images of CaCO_3_-based NIMs taken at different temperatures as shown in figure 10. It is interesting to note that CaCO_3_-based NIMs could not flow completely below the critical temperature, whereas they behaved like a viscous fluid in the absence of solvent at the temperature above the critical points. This phenomenon further confirms the liquid-like behaviours of CaCO_3_-based NIMs.

It has been widely reported that NIMs usually reveal an electrically conductive feature owing to the two reasons as follows: (i) the ion pairs on the macromolecular canopy of nanoparticles can supply charge carriers for NIMs; and (ii) the amorphous macromolecular canopy can provide good passages for the ionic motion [5]. In general, the electrical conductivity of NIMs follows the Vogel−Tammann−Fulcher (VTF) equation given as [38]

3.1 $$\sigma =AT^{-0.5}{\text{e}}^{-B/(T-T_{0})},$$

where *σ* is the conductivity, *T* is the absolute temperature, and *A*, *B* and *T*_0_ are the constants reflecting the relationship between the conductivity and temperature. By combining with the Arrhenius expression, this equation can be rewritten as [39,40]

**Figure 11.**
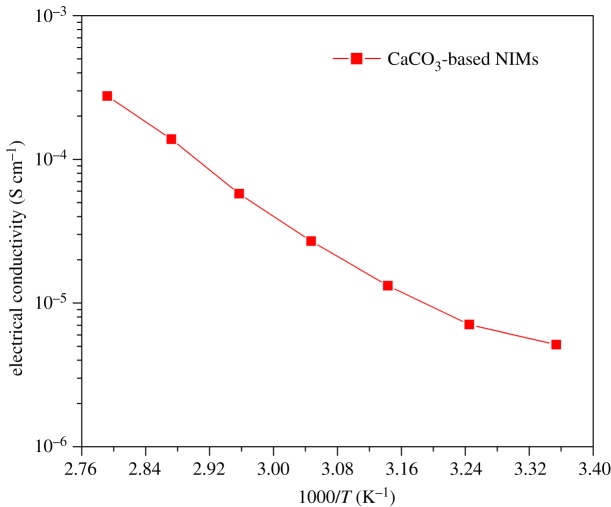
Logarithmic plot of *σ* versus 1000/*T* for CaCO_3_-based NIMs.

3.2 $$\sigma =AT^{-1}{\text{e}}^{-E_{a}/RT},$$

where *E*_a_ is the activation energy and *R* is the Boltzmann constant. Figure 11 shows the logarithmic plot of *σ* versus 1000/*T* derived from the conductive measurement for CaCO_3_-based NIMs at different ambient temperatures, and the activation energy can be determined as 2.261 × 10^3^ eV according to equation (3.2). This plot clearly indicates that the CaCO_3_-based NIMs exhibit proper conductivity in the range of 10^−6^−10^−4^ S cm^−1^. Moreover, the conductivity of CaCO_3_-based NIMs also presents a temperature dependency. It is found the CaCO_3_-based NIMs have a conductivity of 5.1 × 10^−6^ S cm^−1^ at 25°C, but their conductivity increases to 2.7 × 10^−4^ S cm^−1^ at 85°C. These results are better than the conductive data reported by Li *et al*. [30] Therefore, the evaluation of electrical conductivity testifies that the CaCO_3_-based NIMs developed by this work achieved an effective ionic conductivity from both sulfonate anions and quaternary ammonium cations. This gives the CaCO_3_-based NIMs a potential application as electrolyte additives for the lithium-ion battery.

## 4. Conclusion

The CaCO_3_-based NIMs were synthesized via an *in situ* formation method to form the PQAC-CaCO_3_ nanoparticles, followed by an ionic exchange reaction to fabricate an NPEP canopy, and their chemical compositions were confirmed by FTIR spectroscopy and solid-state ^13^C NMR spectroscopy. The CaCO_3_-based NIMs exhibited a rhombohedral shape with a well-defined core-shell structure according to TEM observation, and they also obtained the NPEP canopy with a thickness of 4−6 nm. XRD investigation confirmed that the CaCO_3_ inner core had a calcite crystalline structure, whereas the NPEP canopy was amorphous. The NPEP canopy exhibited a characteristic crystallization–melting behaviour, in which the cold crystallization temperature and melting temperature were both improved in comparison with pristine NPEP owing to the ion-bonding effect. The CaCO_3_-based NIMs also achieved a high loading of the NPEP canopy with a weight fraction of 60.35 wt%. Most of all, the CaCO_3_-based NIMs demonstrated a solvent-free liquid-like behaviour at a temperature above a critical point of 7.8°C. In addition, the CaCO_3_-based NIMs showed a relatively high electrical conductivity with a temperature dependency owing to the ionic conductive effect.
